# *Chlamydia trachomatis* induces disassembly of the primary cilium to promote the intracellular infection

**DOI:** 10.1371/journal.ppat.1012303

**Published:** 2024-06-17

**Authors:** Roseleen Ekka, Abraham Gutierrez, Kirsten A. Johnson, Ming Tan, Christine Sütterlin

**Affiliations:** 1 Department of Developmental and Cell Biology, University of California, Irvine, California, United States of America; 2 Department of Microbiology and Molecular Genetics, University of California, Irvine, California, United States of America; 3 Department of Medicine, University of California, Irvine, California, United States of America; Duke University School of Medicine, UNITED STATES

## Abstract

*Chlamydia trachomatis* is a clinically important bacterium that infects epithelial cells of the genitourinary and respiratory tracts and the eye. These differentiated cells are in a quiescent growth state and have a surface organelle called a primary cilium, but the standard *Chlamydia* cell culture infection model uses cycling cells that lack primary cilia. To investigate if these differences are relevant, we performed infections with host cells that have a primary cilium. We found that *C*. *trachomatis* caused progressive loss of the primary cilium that was prevented by disrupting Aurora A (AurA), HDAC6 or calmodulin, which are components of the cellular cilia disassembly pathway. Stabilization of the primary cilium by targeting this pathway caused a large reduction in infectious progeny although there were no changes in chlamydial inclusion growth, chlamydial replication or the ultrastructural appearance of dividing and infectious forms (RBs and EBs, respectively). Thus, the presence of a primary cilium interfered with the production of infectious EBs at a late step in the developmental cycle. *C*. *trachomatis* infection also induced quiescent cells to re-enter the cell cycle, as detected by EdU incorporation in S-phase, and *Chlamydia*-induced cilia disassembly was necessary for cell cycle re-entry. This study therefore describes a novel host-pathogen interaction in which the primary cilium limits a productive *Chlamydia* infection, and the bacterium counteracts this host cell defense by activating the cellular cilia disassembly pathway.

## Author summary

In this study, we describe a novel tussle between the pathogenic bacterium *Chlamydia trachomatis* and its infected host cell. This interaction is centered on the primary cilium, which is a protrusion on the cell surface that is important for many cellular functions. While the primary cilium is present on cells infected by *Chlamydia* during a natural infection, it is absent from dividing cells used to study *Chlamydia* infections in cell culture. We found that *Chlamydia* infection caused loss of the primary cilium by activating the cellular cilia disassembly machinery and induced a quiescent host cell to re-enter the cell cycle. Furthermore, we showed that the presence of a primary cilium severely inhibited the production of infectious chlamydial progeny. Thus, the host cell uses the primary cilium to restrict a productive infection, while *Chlamydia* induces cilia disassembly as a countermeasure. This host-pathogen interaction could be unique to *Chlamydia*, but it could also have been missed for other intracellular bacteria and viruses, which are commonly studied in cells that lack a primary cilium. This work could lead to new antimicrobial approaches targeting the primary cilium.

## Introduction

*Chlamydia trachomatis* is an important human pathogen that causes a range of infections at different sites in the body [1]. This bacterial species is categorized into serovars, based on antigenic differences that correlate with tissue tropism. Serovars A, B and C cause trachoma, an eye infection that is the leading infectious cause of blindness worldwide [2]. Serovars D-K cause urogenital infections that are the most common cause of bacterial sexually transmitted infection (STI) in the world [3]. Serovar L causes a more systemic STI called lymphogranuloma venereum. To underscore the prevalence of *Chlamydia* infections, almost two-thirds of all reported infections in the U.S. are *C*. *trachomatis* urogenital infections [4]. These chlamydial infections disproportionally affect female reproductive health, including increased risks of tubal infertility and ectopic pregnancy, and seroepidemiologic associations with cervical and ovarian cancer [5,6]. In addition, infants born to mothers infected with *C*. *trachomatis* can develop neonatal pneumonia and conjunctivitis [7].

*C*. *trachomatis*, like all *Chlamydia* spp., is an obligate intracellular bacterium that only replicates within a eukaryotic host cell. The basis for tissue tropism to the eye, or urogenital or respiratory tracts, is incompletely defined, but a common feature is that *Chlamydia* predominantly infects epithelial cells, which are terminally differentiated and therefore quiescent [8]. This non-dividing growth state of target cells during a natural infection is in stark contrast to the standard cell culture model of *Chlamydia* infection, which uses cycling cells from human and animal cell lines that are relatively easy to grow and produce high infectious yields of chlamydiae [9]. These cell culture infection studies have allowed the elucidation of many *Chlamydia*-host interactions [10], including effects on host organelles, such as the Golgi apparatus, endoplasmic reticulum, mitochondria and centrosome [11–16]. However, it is unknown if the growth state of the host cell affects interactions with organelles and the intracellular infection.

A distinctive feature of differentiated cells in the body is the presence of a single primary cilium on their apical cell surface. For example, the cells lining the endometrium and the fallopian tube at sites of a natural *C*. *trachomatis* infection have a primary cilium [17–19]. In contrast, the cycling cells typically used for *Chlamydia* and other cell culture infection models lack these prominent cell surface protrusions. The primary cilium is an important signaling organelle that is required for signal transduction through multiple different pathways [20]. This microtubule-based cell surface protrusion extends from the mother centriole of the centrosome, which is then known as the basal body. The primary cilium is a highly dynamic structure that forms when a cell exits the cell cycle and undergoes regulated disassembly upon cell cycle re-entry in response to growth signals [21,22]. Primary cilia and cell cycle progression are intimately linked because the primary cilium sequesters the centrosome to the plasma membrane and limits the ability of a cell to progress through mitosis, which is when the centrosome forms the spindle poles [23]. A central pathway that controls cilia disassembly involves the mitotic kinase Aurora A, whose activation at the basal body requires Ca^2+^/calmodulin [24]. Activated Aurora A phosphorylates its effector HDAC6 [22], which promotes cilia disassembly by deacetylating and destabilizing axonemal microtubules and by deacetylating cortactin to reorganize the actin cytoskeleton [21,22,25].

In this study, we investigated the relevance of the primary cilium for *Chlamydia* infection by using host cells that have been induced to from a primary cilium. We report that *C*. *trachomatis* caused loss of the primary cilium via the host AurA-HDAC6 cilia disassembly pathway and induced quiescent cells to re-enter the cell cycle. We also demonstrate that the presence of the primary cilium had a severe negative effect on a productive *C*. *trachomatis* infection, providing a rationale for why *Chlamydia* induces primary cilia loss. These studies reveal that the primary cilium is an important battleground between *C*. *trachomatis* and its host cell.

## Results

### *C*. *trachomatis* causes loss of the primary cilium

**Fig 1:**
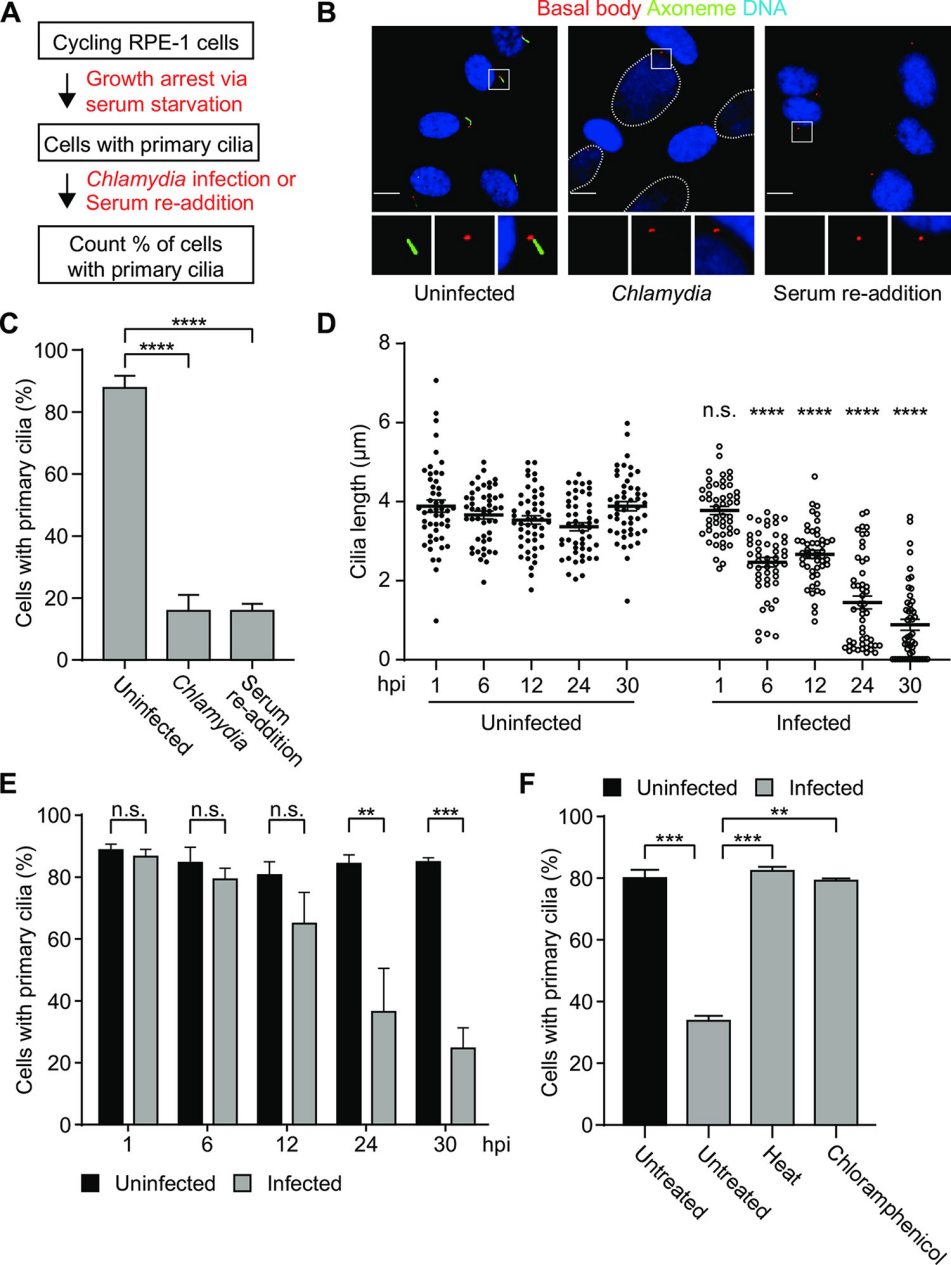
*Chlamydia* causes progressive cilia disassembly. (A) Schematic representation of the experimental design. (B) RPE-1 cells, grown in serum-free medium for 48 hours to induce primary cilia formation, were subjected to infection with *C*.*trachomatis* L2 or serum re-addition. At 30 hours post infection (hpi), cells were fixed and processed for immunofluorescence microscopy analysis with antibodies to Arl13b to stain the ciliary axoneme (green), Cep164 to detect the basal body (red) and NucBlue to visualize the nucleus (blue). Dashed lines indicate chlamydial inclusions. Insets show magnified views of the boxed area. Scale bar: 10 μm. (C) Quantification of the images in B. The percentage of cells with primary cilia is shown. (D) The length of cilia from > 50 uninfected or infected cells grown in serum-free medium were measured at the indicated time points for uninfected vs infected cells. The average cilia length is shown. At each time point, the length of cilia from infected cells was compared to that of uninfected cells and analyzed by an unpaired, two-tailed t test. ns: non-significant, ****P<0.0001, *** *P*<0.001. (E) The percentage of cells with primary cilia is shown at each time point for uninfected and infected cells grown in serum-free medium. (F) RPE-1 cells, grown in serum-free medium, were infected with *C*.*trachomatis* EBs that were either left untreated, heat inactivated for 10 min at 56°C or incubated with 70 μg/ml chloramphenicol. Cells were fixed at 30 hpi and processed for immunofluorescence microscopy analysis. The percentage of cells with cilia is shown. For each graph in this figure, three independent biological replicates were performed. Data is presented as mean ± SEM. Data were analyzed with an unpaired, two-tailed t test. ns: non-significant, ****P<0.0001, *** *P*<0.001, ** *P*<0.01. See also S1 Fig.

To investigate the role of the primary cilium, we modified the standard *Chlamydia trachomatis* cell culture infection model [9] by using quiescent epithelial cells as the host cell. Following established protocols, dividing cells were serum starved cells for 48 hours to cause growth arrest and induce primary cilia formation. These quiescent cells were then infected with *C*. *trachomatis* serovar L2 and maintained without serum for the duration of the infection (Fig 1A).

*Chlamydia* infection induced loss of the primary cilium in four cell lines that were tested. For example, only 16% of infected RPE-1 (human retinal epithelial cells) had a primary cilium at 30 hours post infection (hpi), compared to 88% of uninfected RPE-1 cells (Fig 1B and 1C). Similarly, *C*. *trachomatis* caused primary cilia loss in HeLa cells and three murine cell lines (IMCD3, NIH 3T3 cells and mouse embryonic fibroblasts (MEFs, S1A Fig). RPE-1 cells are diploid, non-transformed cells that are commonly used for primary cilia studies [21,22]. As RPE-1 cells readily form primary cilia when growth arrested through serum starvation, we used them for the rest of our cilia studies.

We examined other features of *Chlamydia*-induced cilia loss in additional experiments. We first verified that growth-arrested and dividing RPE-1 cells had similar infectivity, as measured by the percentage of cells with chlamydial inclusions (S1B Fig). *Chlamydia*-induced cilia loss was similar to the effect of serum re-addition (Fig 1B and 1C), which induces cilia disassembly by promoting cell cycle re-entry [22]. Loss of the primary cilium in *Chlamydia*-infected cells was progressive, with shortening of the ciliary axoneme beginning at 6 hpi (Figs 1D and S1C) and reduced prevalence of ciliated cells starting at 24 hpi (Fig 1E). It required an active infection because there was no cilia loss with heat-inactivated or chloramphenicol-treated chlamydiae (Fig 1F). *Chlamydia*-induced cilia loss also occurred when we induced primary cilia formation via contact inhibition in mouse embryonic fibroblasts (S1D Fig). We also showed that a different chlamydial strain, *C*. *trachomatis* serovar D, caused cilia loss during infection of RPE-1 cells (S1E Fig). These data demonstrate that *Chlamydia trachomatis* infection causes a mammalian host cell to lose its primary cilium independent of the cell line or serovar, or the method used to induce cilia formation.

### *Chlamydia*-induced cilia loss requires the host AurA-HDAC6 cilia disassembly pathway

**Fig 2:**
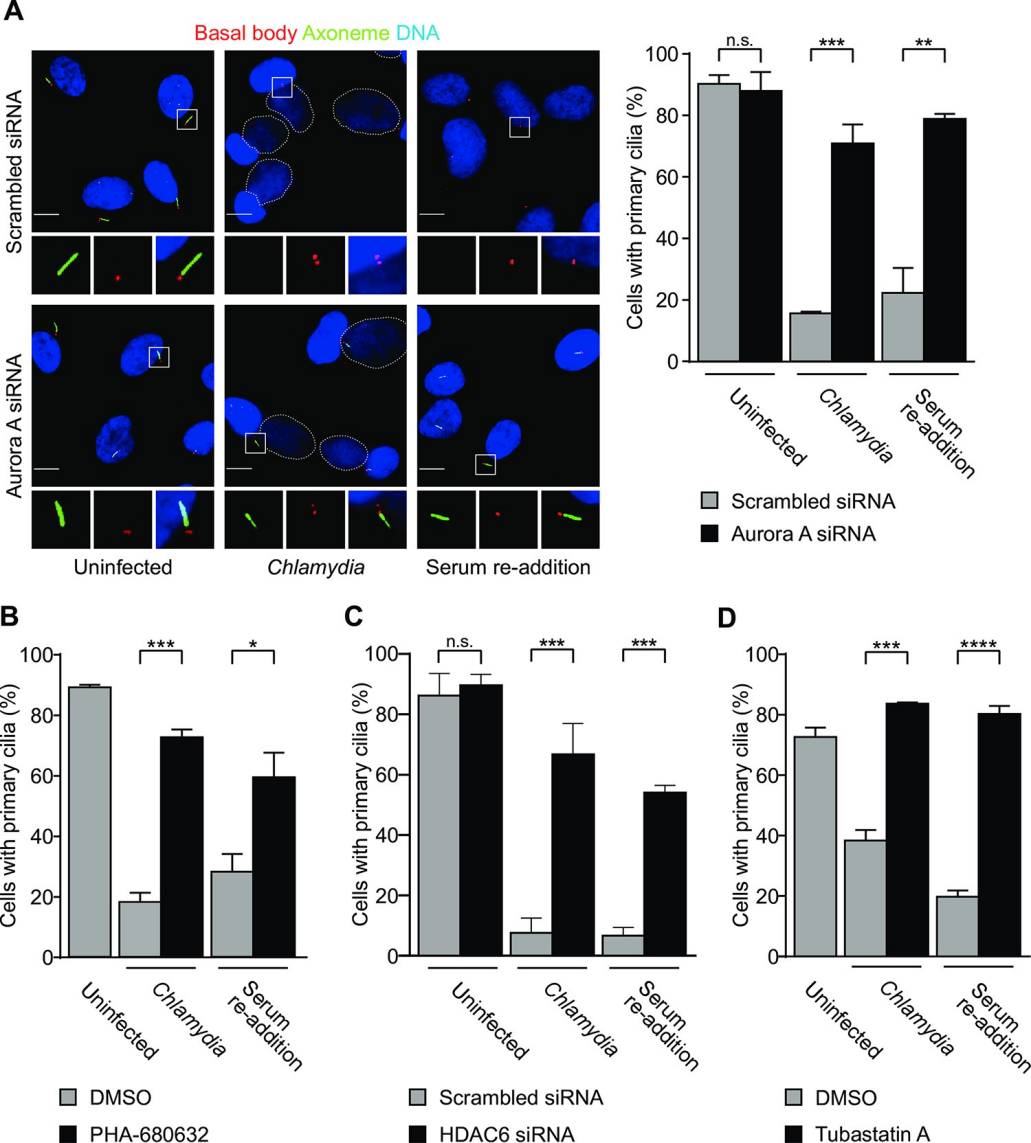
Aurora A and HDAC6 are both necessary for *Chlamydia*-induced cilia disassembly. (A) RPE-1 cells were transfected with either scrambled or AurA-specific siRNA for 48 hours in serum-free medium, followed by either infection with *C*.*trachomatis* L2 or serum re-addition. At 30 hpi, cells were fixed and processed for immunofluorescence microscopy with antibodies to the ciliary axoneme (Arl13b) and the basal body (Cep164). DNA was visualized with the DNA dye NucBlue. Dashed lines indicate chlamydial inclusions. Insets show magnified views of the boxed regions. Scale bars: 10 μm. The percentage of cells with primary cilia is shown. (B) RPE-1 cells, grown in serum-free medium, were treated with either DMSO or PHA-680632 for 2 hours prior to infection with *C*.*trachomatis* L2 or serum re-addition. After 30 hpi, cells were processed for immunofluorescence microscopy with antibodies to the ciliary axoneme and the basal body. The percentage of cells with primary cilia is shown. (C) Same as in (A) except for HDAC6 depletion by siRNA. The percentage of scrambled and HDAC6-depleted cells with primary cilia is shown. (D) Same as in (B) except for the use of the HDAC6-specific inhibitor tubastatin A (5 μM). The percentage of cells with primary cilia is shown. For each graph in Fig 2, three independent biological replicates were performed. Data is presented as mean ± SEM. Data were analyzed using an unpaired, two-tailed t test. ns: non-significant, ****P<0.0001, *** *P*<0.001, ** *P*<0.01. See also S2 and S3 Figs.

The similarity of primary cilia loss induced by serum re-addition and *Chlamydia* infection prompted us to examine whether *Chlamydia* causes primary cilia loss via the cellular AurA-HDAC6 cilia disassembly pathway. When we depleted AurA with siRNA (S2A Fig), 71% of *Chlamydia*-infected cells had normal-appearing primary cilia, compared to 15.6% in a scrambled siRNA control (Fig 2A). This protective effect of AurA depletion was similar to what is reported for serum re-addition [22] and was further confirmed with the small molecule inhibitor of AurA, PHA-680632 (Fig 2B). Cilia disassembly, induced by either *C*. *trachomatis* or serum re-addition was also prevented by disruption of HDAC6, by siRNA (Figs 2C and S2C) or with the specific inhibitor tubastatin A (Fig 2D), and by inhibition of the AurA regulator calmodulin with the inhibitor CMZ (S3A and S3B Fig). These studies provide strong evidence that *C*. *trachomatis* induces loss of the primary cilium through the cellular AurA-HDAC6 pathway.

### *Chlamydia* infection does not affect primary cilia formation

We next investigated if *C*. *trachomatis*-induced cilia loss is also due to a defect in cilia formation and maintenance. For these studies, we infected cycling RPE-1 cells that lack a primary cilium and induced cilia formation while blocking cilia disassembly with the HDAC6 inhibitor tubastatin A (S4A Fig). With this experimental set-up, uninfected control cells readily formed cilia, and addition of tubastatin A slightly increased the prevalence of ciliated cells because cilia disassembly was prevented (S4B and S4C Fig). With infection, primary cilia were only present on 38% of cells, consistent with *Chlamydia*-induced cilia disassembly, but 73% of cells had primary cilia when cilia disassembly was prevented with tubastatin A (S4B and S4C Fig). These results indicate that infected cells retain the ability to form and maintain a primary cilium. Thus, *C*. *trachomatis* causes cilia loss by promoting cilia disassembly and not by affecting cilia formation or maintenance.

### Cilia disassembly is necessary for a productive infection

**Fig 3:**
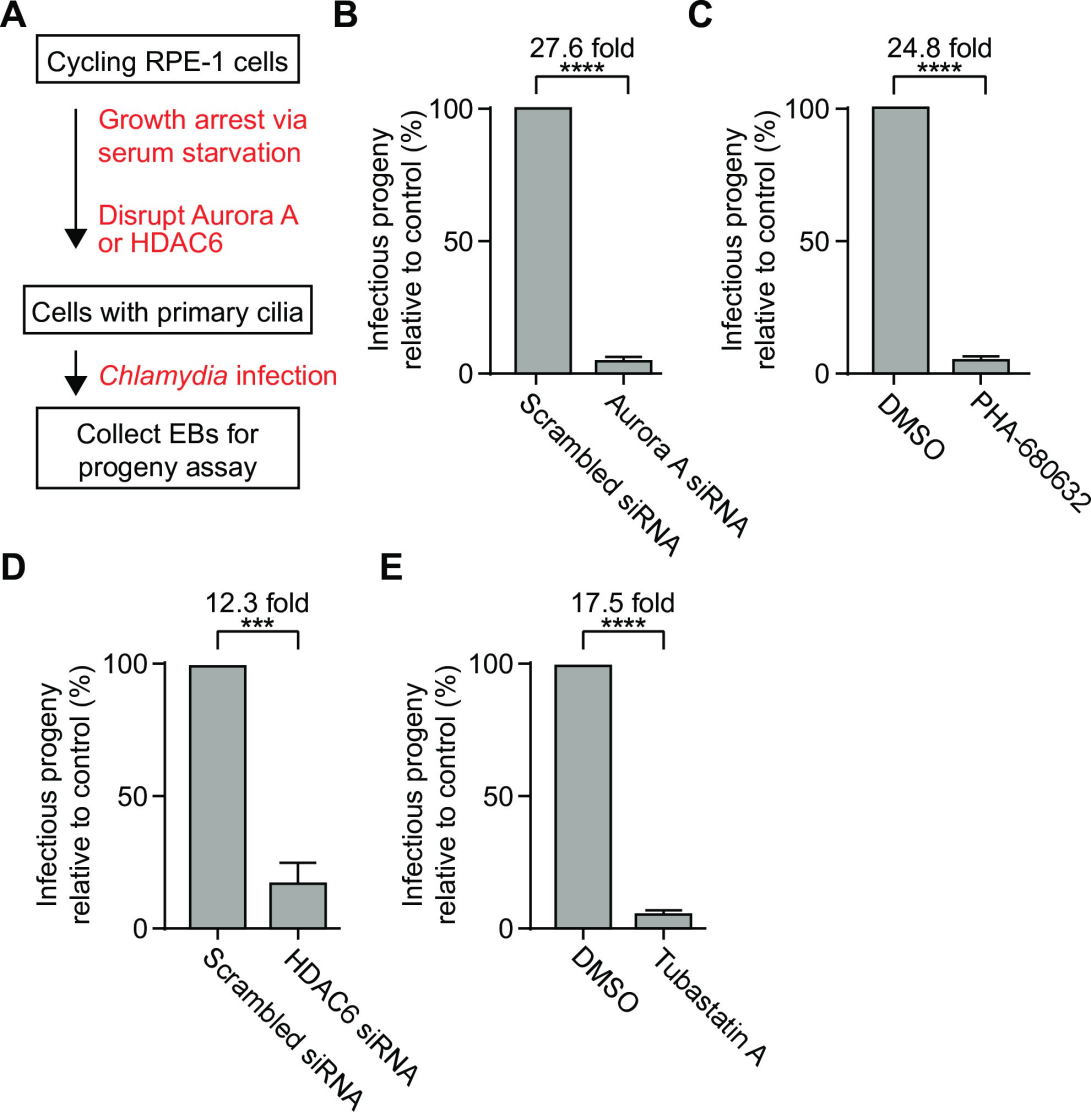
Cilia disassembly is necessary for a productive infection. (A) A schematic representation of the experimental design is shown. (B) RPE-1 cells were transfected with either scrambled or AurA-specific siRNA for 48 hours in serum-free medium, followed by infection with *C*. *trachomatis* L2. At 30 hpi, cells were lysed, and lysates were used to determine the number of infectious progeny through secondary infections. The fold change between scrambled and AurA-depleted cells is shown. (C) RPE-1 cells, grown in serum-free medium for 48 hours, were incubated with DMSO or 0.5 μM of PHA-680632 for 2 hours prior to infection with *C*.*trachomatis* L2. After 30 hours of incubation in absence or presence of PHA-680632, cells were lysed, and lysates were used for secondary infections to determine the number of infectious progeny. The fold change between DMSO and PHA-680632-treated cells is also shown. (D) Same as in (B), except that scrambled and HDAC6-depleted cells were analyzed. (E) Same as in (C), except that DMSO and tubastatin A-treated cells were analyzed. For each graph in Fig 3, three independent biological replicates were performed. Data is presented as mean ± SEM. An unpaired two-tailed t test was performed to compare the progeny after various manipulations with their respective controls. ns: non-significant, ****P<0.0001, *** *P*<0.001, ** *P*<0.01.

Our ability to prevent cilia loss by disrupting the AurA-HDAC6 cilia disassembly pathway provided a means to investigate the effect of a primary cilium on the *Chlamydia* infection (Fig 3A). For these experiments, we assessed the impact of cilia stabilization with a progeny assay, which is a standard assay to measure the production of infectious chlamydiae in a secondary infection [9]. AurA depletion with siRNA caused a 27.6-fold decrease in infectious progeny compared to control cells treated with scrambled siRNA (Fig 3B). Consistent with this result, inhibition of AurA with PHA-680632 reduced infectious progeny by 24.8-fold, compared to a DMSO-treated control infection (Fig 3C). Disruption of HDAC6 with siRNA or tubastatin A reduced infectious progeny by 12.3-fold and 17.5-fold, respectively (Fig 3D and 3E), and inhibition of calmodulin with CMZ decreased infectious progeny by 23.7-fold (S3C Fig). Thus, preventing cilia disassembly by targeting three separate components of the AurA-HDAC6 pathway produced a consistent and severe progeny defect. These findings demonstrate that the presence of a primary cilium on the host cell restricts a productive *Chlamydia* infection.

### Cilia stabilization does not alter inclusion growth, chlamydial replication or the ultrastructural appearance of RBs and EBs

**Fig 4:**
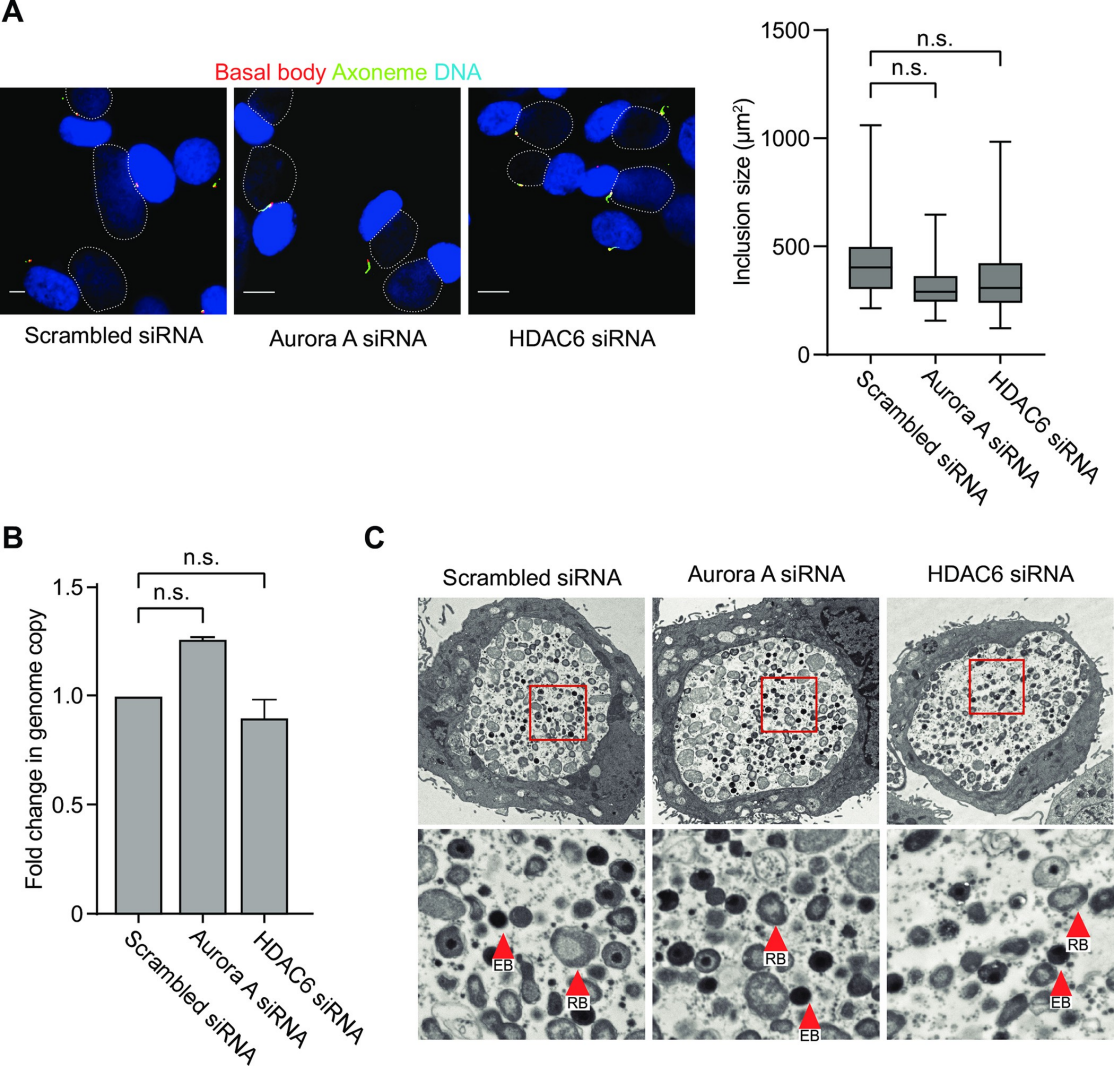
Cilia stabilization does not alter replication and ultrastructural appearance of developmental forms. (A) RPE-1 cells that were grown in serum-free medium and at the same time transfected with either scrambled or AurA or HDAC6-specific siRNA for 48 hours, and infected *C*. *trachomatis* L2. At 30 hpi, cells were fixed and processed for immunofluorescence analysis with antibodies to the ciliary axoneme (Arl13b) and the basal body (Cep164). DNA was labeled with NucBlue which was used to detect the size of inclusion (marked with dotted white lines). Infected cells with stabilized and disassembled cilia are shown. Scale bar: 10 μm. The size of > 100 inclusions from >15 areas of view from 3 biological replicates was measured. A box plot is shown with mean inclusion size indicated as a line. A 2-way ANOVA with multiple comparisons was performed to analyze the inclusion size. ns: non-significant. (B) The cells described in (A), were also analyzed by qPCR to determine the number of chlamydial genomes. The fold change between scrambled and AurA or HDAC6 siRNA-treated cells is shown. The date was analyzed with an unpaired two-tailed t test. ns: non-significant, ****P<0.0001, *** *P*<0.001, ** *P*<0.01. (C) EM analysis of infected cells treated with scrambled, AurA and HDAC6-specific siRNA. Representative images are shown. A magnified view of the boxed areas is also shown for each sample. RBs and EBs are indicated with arrow heads. In the same experiment as this EM analysis, we observed a 25-fold and 33-fold reduction in infectious progeny for AurA- or HDAC6-depleted cells, respectively, when compared to scrambled control cells.

To understand how cilia stabilization caused a defect in infectious progeny production, we performed additional analyses of the *Chlamydia* developmental cycle. For these experiments, we prevented primary cilia loss by using siRNA to separately deplete AurA or HDAC6. Immunofluorescence analysis showed no difference in the size of the chlamydial inclusion, which is the membrane-bound compartment where chlamydiae reside within an infected host cell (Fig 4A). Using a qPCR genome copy assay, we found that neither AurA nor HDAC6 knockdown had an effect on the number of chlamydiae at 30 hpi (Fig 4B). Examination of inclusions by electron microscopy (EM) revealed that these inclusions contained a mix of the two main developmental forms (RBs, which are the replicating form, and EBs, which are the infectious form) that was similar in knockdown and scrambled siRNA control cells (Fig 4C). Together, these results provide evidence that the presence of a primary cilium does not prevent inclusion growth, RB replication or EB production. An important consideration is that this EM analysis only reveals the ultrastructural appearance of EBs and does not measure if they are infectious.

### Aurora A is not necessary for *Chlamydia* infection in cells without primary cilia

**Fig 5:**
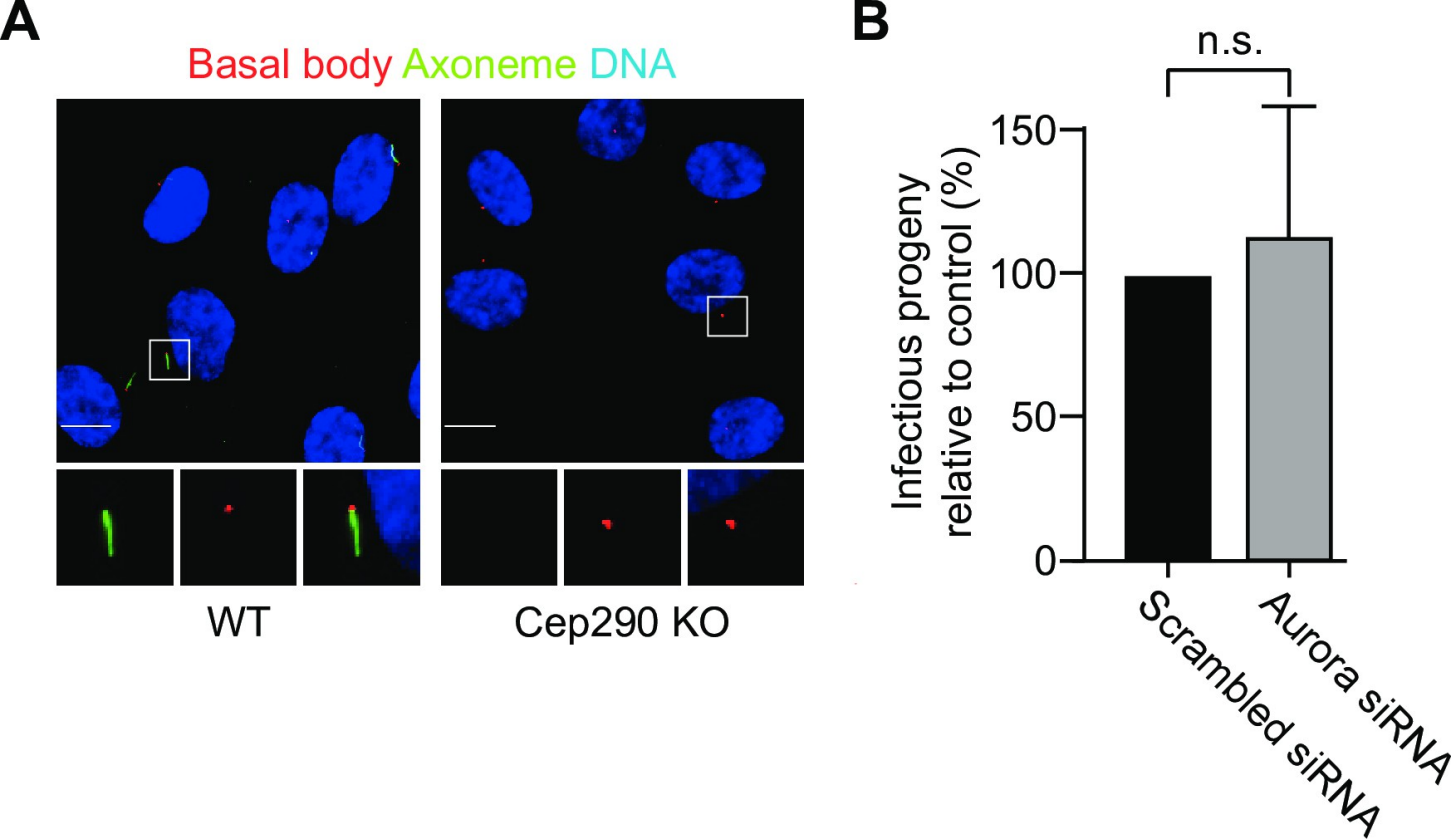
Aurora A is not necessary for *Chlamydia* infection in cells without cilia. (A) RPE-1 WT and Cep290 KO cells were serum starved for 48 hours, fixed and processed for immunofluorescence microscopy with antibodies Arl13b to stain the ciliary axoneme (green), Cep164 to detect the basal body (red) and NucBlue to visualize the nucleus (blue). Insets show magnified views of the boxed regions. Scale bar: 10 μm. (B) Cep290 KO cells were transfected with either scrambled or AurA-specific siRNA for 48 hours in serum-free medium, followed by infection with *C*. *trachomatis* L2. At 30 hpi, cells were lysed, and lysates were used to determine the number of infectious progeny through secondary infections. Infectious progeny relative to control is shown. An unpaired t-test was performed. ns: non-significant. See also S4D and S5 Figs.

We next investigated if the AurA pathway is required to promote cilia disassembly or, alternatively, to control a cilia-independent function of pathway components during the infection. To distinguish between these possibilities, we repeated our AurA disruption experiments with host cells that cannot form a primary cilium. We used Cep290 KO RPE-1 cells that are unable to form cilia (Fig 5A) because they lack the basal body-associated ciliogenesis factor Cep290 [26,27]. We first verified that *C*. *trachomatis* serovar L2 infects Cep290 KO cells (S5A Fig) and produces infectious progeny (S5B Fig) similar to WT RPE-1 cells. We then found that Cep290 KO RPE-1 cells treated with AurA siRNA produced infectious progeny at levels comparable to control cells (Fig 5B). These results demonstrate that AurA was no longer required if the *Chlamydia* infection was performed with host cells without primary cilia. Thus, AurA appears to only be necessary to mediate cilia disassembly during a *C*. *trachomatis* infection.

### *Chlamydia*-induced cilia loss is necessary for cell cycle re-entry

**Fig 6:**
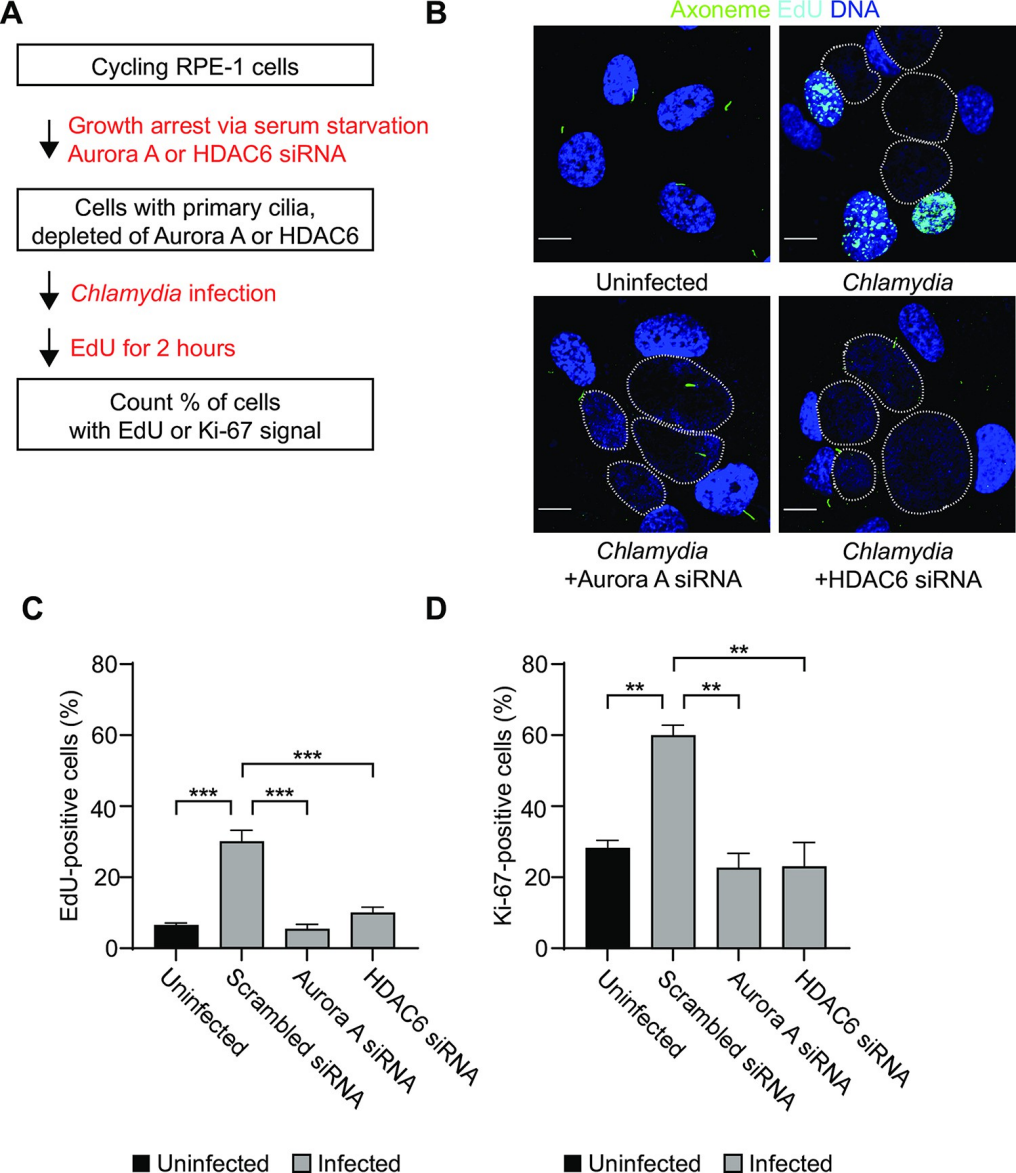
*Chlamydia*-induced cilia loss is necessary for cell cycle re-entry. (A) A schematic representation of the experimental design is shown. (B) RPE-1 cells were left untreated or transfected with either scrambled, AurA or HDAC6-specific siRNA for 48 hours in serum-free medium, followed by infection with *C*.*trachomatis* L2 at MOI 3. After 34 hours, cells were labeled with EdU for 2 hours using click chemistry (shown in cyan) and then processed for immunofluorescence microscopy with antibody to Arl13b (ciliary axoneme). DNA was labeled with the DNA dye NucBlue, and inclusions are marked with dotted white lines. Representative images are shown for each condition. Scale bar: 10 μm. (C) A quantification of the percentage of EdU-positive cells from (B) is shown. (D) Same as in (B), but cells were fixed at 36 hpi and processed for immunofluorescence microscopy with antibodies to the Ki-67 proliferation marker. The percentage of Ki-67-positive cells are shown. For each graph in Fig 6, three independent biological replicates were performed. Data is presented as mean ± SEM. A 1-way ANOVA with multiple comparisons was performed for (C) and (D). ns: non-significant, ****P<0.0001, *** *P*<0.001, ** *P*<0.01. See also S6 Fig.

As primary cilia disassembly and cell cycle re-entry are physiologically linked, we examined if *Chlamydia*-infected cells undergoing primary cilia loss also re-enter the cell cycle. We first used fluorescence microscopy to assay EdU incorporation, which detects DNA synthesis in S-phase as a measure of cell cycle re-entry (Fig 6A). *C*. *trachomatis* infection increased EdU-positive cells from 6% to 30% during a 2 hour incubation window (Fig 6B and 6C). This level of cell cycle re-entry was lower than observed with serum re-addition (S6B and S6C Fig). *Chlamydia*-induced cell cycle re-entry was prevented when we stabilized the primary cilium by disrupting the AurA-HDAC6 pathway with siRNA and inhibitors (Figs 6B, 6C and S6E). We obtained similar data with the proliferation marker Ki-67 (Figs 6D and S6D). These results demonstrate that *C*. *trachomatis* induces a quiescent host cell to disassemble its primary cilium and re-enter the cell cycle.

**Fig 7:**
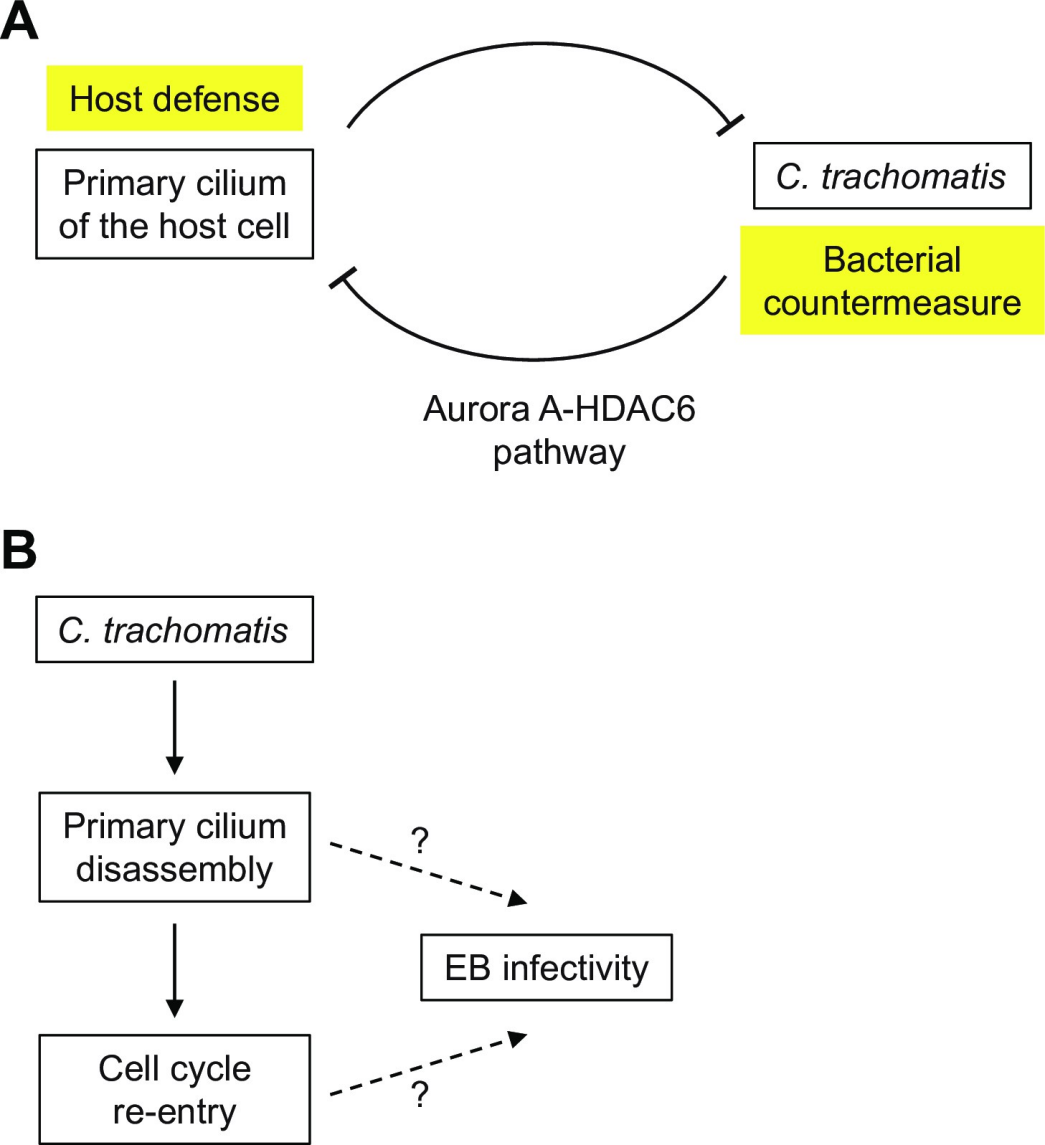
Models for antagonistic relationship between a quiescent host cell with primary cilium and *Chlamydia trachomatis*. (A) The primary cilium on the cell surface of the host cell limits the chlamydial infection, but *C*.*trachomatis* avoids this host cell defense by inducing primary cilium disassembly through activation of the AurA-HDAC6 pathway. (B) Proposed model for the regulation of EB infectivity, which could be determined by primary cilium disassembly, cell cycle re-entry or both.

In summary, our study has revealed a novel antagonistic interaction between *Chlamydia* and its quiescent host cell (Fig 7A), in which the host cell uses its primary cilium to limit the infection at a late stage of the bacterial developmental cycle. However, *Chlamydia* counteracts this host defense mechanism by activating the AurA-HDAC6 pathway, which leads to cilia disassembly. We have also shown that *Chlamydia* infection induces cell cycle re-entry, which can be prevented by blocking cilia disassembly. Future studies will be necessary to determine if the production of infectious progeny is promoted by cilia disassembly or cell cycle re-entry, or by both processes together (Fig 7B).

## Discussion

In this study, we demonstrate that the primary cilium is necessary for a novel host defense against *C*. *trachomatis* and also the target of a chlamydial countermeasure. To more closely mimic a natural infection of quiescent, differentiated host cells, we adapted the standard *Chlamydia* cell culture model by using host cells that had been growth arrested to induce primary cilia formation. This approach revealed that *C*. *trachomatis* infection caused loss of the primary cilium by enhancing the cellular AurA-HDAC6 cilia disassembly pathway and induced cell cycle re-entry. Infectious progeny production was greatly reduced when we stabilized the primary cilium, providing evidence that the primary cilium has a novel anti-chlamydial effect, either directly or mediated by its known role in controlling the cell cycle.

The use of human and animal cell lines for *Chlamydia* cell culture infections has been invaluable for uncovering many host-*Chlamydia* interactions [10]. Up to the 1960s, *Chlamydia* was cultivated in embryonated chicken eggs, which contain differentiated epithelial cells [28,29]. In comparison, cell lines were a major innovation that provided a convenient and easily expandable source of host cells and produced up to ten times higher infectivity [30,31]. Interestingly, chlamydial strains adapted to cell culture growth often lost their ability to infect egg yolks [30]. This observation suggests that long-term passage of chlamydiae in cycling cells that lack a primary cilium may cause a laboratory strain to lose the ability to cause cilia loss. However, the strains that we used in this study (serovar L2/434/Bu and serovar D/UW-3/Cx) have been passaged in cell culture off and on for twenty years [9,32] and yet have retained their ability to induce cilia disassembly.

Our study demonstrates that *C*. *trachomatis* causes an infected host cell to lose its primary cilium by enhancing cilia disassembly. Using knockdown and pharmacological approaches, we found that multiple components of the cellular AurA-HDAC6 cilia disassembly pathway are necessary for *Chlamydia*-induced cilia loss. Our experiments with heat-inactivated and chloramphenicol-treated EBs indicate that an active infection and de novo chlamydial protein production are required to cause primary cilia loss. The most likely model is that a chlamydial factor interacts with a component of the cilia disassembly pathway to enhance its activity or promote its recruitment to the primary cilium. For a putative chlamydial factor to be able to interact with these cytosolic host factors, it must have physical access, either by being secreted into the host cytosol or into the membrane of the chlamydial inclusion, which is the compartment in which the chlamydial population resides. However, a host interactor screen of chlamydial inclusion membrane proteins did not reveal any components or regulators of the AurA-HDAC6 cilia disassembly pathway [33]. Thus, additional studies will be necessary to identify the chlamydial protein that alters the activity of the cilia disassembly pathway.

Intriguingly, *C*. *trachomatis* infection of host cells with a stabilized primary cilium caused a severe reduction in infectious progeny production, but no other obvious deficit in the intracellular infection. Inclusion growth was normal, as was the developmental cycle as measured by the number and appearance of chlamydiae within the inclusion, including both the replicating (RB) and infectious (EB) forms. This disconnect between the production of normal-looking EBs and a large decrease in infectious progeny suggests that EBs produced in host cells with a primary cilium are not infectious. Such an EB maturation defect has not been previously described, but suggests that the primary cilium exerts its anti-chlamydial effect at a late step in the 48-hour developmental cycle. Further studies will be required to identify this defect in EBs produced in a host cell with a primary cilium.

The primary cilium may mediate its anti-chlamydial effect through several potential mechanisms. It could be exerted through the known function of the primary cilium as a transduction hub for multiple signaling pathways, including Hedgehog, PDGF, Wnt and Notch signaling [34]. Alternatively, it could be related to autophagy, which is regulated by the primary cilium [35] and has been shown to restrict *C*. *trachomatis* growth [36]. The primary cilium could also function as a structural cell cycle checkpoint [37]. We found that *C*. *trachomatis* infection caused a quiescent cell to re-enter the cell cycle, which was blocked by stabilization of the primary cilium. Cell cycle re-entry could be beneficial for the bacterium because the altered growth state of the host cell could allow enhanced access to nutrients and metabolites. There is some precedent, as many DNA viruses favor replication in cycling cells over quiescent cells because of increased deoxynucleotide availability [38,39]. It is not known if cell cycle re-entry is beneficial to *C*. *trachomatis*, and if it is cell cycle re-entry and/or primary cilium disassembly that promotes the generation of infectious EBs.

This is the first report of a microbial pathogen that causes primary cilium loss by enhancing the AurA-HDAC6 cilia disassembly pathway. Interestingly, Sars-CoV-2 induces primary cilia loss, but through a completely different mechanism in which cilia formation and maintenance is disrupted [40]. The viral protein ORF10 activates the ubiquitin ligase CUL2^ZYG11B^, promoting ubiquitination and degradation of IFT46, which is required for primary cilium formation and maintenance. The only reported effect of a bacterium on the primary cilium is a study that showed that treatment of cells with *P*. *aeruginosa* lectin LecB removed primary cilia in the context of major actin reorganization [41]. In both these cases, there was no evidence of AurA-HDAC6 pathway involvement, or that primary cilia loss was beneficial to the microbe. AurA kinase activity has not been linked with bacterial infection, but appears to promote replication of several viruses [42,43]. In addition, the E6 ligase of human papillomavirus (HPV) interacts with AurA to promote HPV-mediated carcinogenesis [27].

In conclusion, we show for the first time that the primary cilium is a battleground between *Chlamydia* and its host cell as well as a new therapeutic target. We demonstrate that *C*. *trachomatis* causes loss of the primary cilium to counter the strong anti-chlamydial effect of this cell surface organelle. This novel host-pathogen interaction has likely been overlooked because the prevailing *Chlamydia* cell culture infection model utilizes dividing cells that do not have a primary cilium. The primary cilium could possibly have an unrecognized role for other intracellular bacteria and viruses that are studied with infection models that use cycling cells lacking primary cilia. Our study also identified a new potential strategy for combating *C*. *trachomatis* infections by targeting the AurA-HDAC6 cilia disassembly pathway. In this regard, small molecule inhibitors of AurA and HDAC6 have been tested in clinical trials for treatment of cancer and neurodegenerative disease [44,45] and could serve as the basis for a novel class of anti-chlamydial antibiotics.

## Material and methods

### Antibodies, siRNA and pharmacological reagents

#### Primary antibodies used in this study

polyclonal rabbit anti-Arl13b (Invitrogen PAS 32035, used for Immunofluorescence analysis (IF) at a dilution of 1:500), polyclonal goat anti-Cep164 (Santa Cruz Biotech Sc 240226, IF:1:500), monoclonal mouse anti-MOMP (gift from Dr. Ellena Peterson, UCI), monoclonal mouse α-Tubulin (Sigma-Aldrich T5168, used for Wester blotting (WB) at a dilution of 1:10,000), polyclonal rabbit anti-Aurora A (Cell Signaling 3092, WB: 1:1000), monoclonal mouse anti-GAPDH (Santa Cruz, sc-47724, WB: 1:2000), polyclonal mouse anti γ-tubulin (Abcam Ab11317, IF: 1:500), Ki-67 (Santa Cruz-BioTechnologies, sc-23900, IF:1:200).

#### Secondary antibodies for immunofluorescence microscopy

Donkey anti-Rabbit IgG Alexa Fluor 488 (Invitrogen, A21206), Goat anti-mouse IgG Alexa Fluor 647 (Invitrogen, A21236), Donkey anti-mouse IgG Alexa Fluor 555 (Invitrogen, A32773) and Donkey anti-Goat IgG Alexa Fluor 594 (Invitrogen, A11058).

#### Secondary antibodies for western blot

Goat anti-rabbit IgG LI-COR IRDYE 680 (926-680-71, Fisher Scientific) and goat anti-mouse IgG LI-COR IRDye 800 (926–32210; Fisher Scientific).

#### siRNAs

ON-TARGET plus Human AURKA (6790) siRNA-Smartpool from Dharmacon, 5nmol), ON-TARGETplus Human HDAC6 (10013) siRNA-individual from Dharmacon (J-003499-05-0005, J-003499-08-0005, 5nmol), Silencer select Negative control No.1 siRNA (Scrambled siRNA) was obtained from Thermofisher scientific (#4390843, 5nmol)

#### Pharmacological reagents

Aurora A kinase inhibition: PHA-680632 (Selleckchem/Fisher Scientific, S1454), calmodulin inhibition: Calmidazolium (CMZ, Calbiochem # 208665), HDAC6 inhibition: Tubastatin A (Sigma-Aldrich, SML0044), chloramphenicol (Acros Organics, ACROS 227921000), Click-iT EdU Cell Proliferation Kit for Imaging, Alexa Fluor 488 dye (CLICK EDU ALEXA 488 IMAGING) (Thermo Fisher Scientific (Life Technologies, C10337).

### Cell culture, transfections and *Chlamydia* infections

#### Cells

HeLa, hTERT-RPE-1, NIH3T3 and mouse embryonic fibroblast cell lines were from ATCC (Manassas, VA), hTERT-RPE-1 GFP-SMO Cep290KO were a generous gift from Dr. Joon Kim (KAIST, South Korea). IMCD3 mouse kidney cells were kindly provided by Dr. Maxence Nachury, UCSF). Cells were grown in DMEM (Invitrogen) supplemented with 10% FBS in a 5% CO_2_ at 37°C.

#### Chlamydia strains

*C*.*trachomatis* serovar L2, strain L2/434/Bu (ATCC VR-902B) and *C*. *trachomatis* serovar D, strain UW-3/Cx (generous gift by Dr. Kevin Hybiske, University of Washington).

#### Chlamydia infection

Cell monolayers at 70% confluency were infected with *C*. *trachomatis* serovar L2 (L2/434/Bu), or serovar D, at a multiplicity of infection (MOI) of 3 in sucrose-phosphate-glutamic acid (SPG) buffer (200mM sucrose,20mM sodium phosphate anhydrous dibasic, 5mM glutamate, pH 7.2) by centrifugation at 700 x g in an Eppendorf 5810 R centrifuge for 1 hr. NIH3T3 (70% confluency) were infected in serum-free DMEM and centrifuged at 300 x g for 30 minutes. Uninfected control cells were subjected to mock infections in SPG or DMEM. Inoculum was replaced with fresh cell culture medium and monolayers were incubated at 37°C in 5% CO_2_ for the time indicated.

#### Knockdown transfections

Transient transfection of hTERT-RPE-1 and Cep290 KO cells with scrambled, Aurora A or HDAC6 siRNAs was carried out using Oligofectamine (Invitrogen), according to the manufacturer’s recommendations. After the transfection, they were grown for 48 hours in serum-free medium and then either infected with *C*.*trachomatis* or subjected to re-addition of 10% serum. Cells on coverslips were fixed and processed for immunofluorescence and in parallel harvested for western blot analysis.

### Immunofluorescence microscopy

Cells grown on coverslips were fixed in ice-cold 100% methanol for 5–7 min, permeabilized and blocked in saponin blocking buffer (0.1% saponin, 2% FBS, 0.5%NaN3 in PBS) for 1h at RT. Cells were then stained with primary antibodies in saponin blocking buffer for 1hr at RT, followed by washing with saponin buffer and incubation with secondary antibodies conjugated to either Alexa 488, Alexa 594 or Alexa 647 (Invitrogen) for 1 hour. Coverslips were washed 3 times with PBS and mounted on glass slides using prolong glass antifade with NucBlue (Sigma-Aldrich). Immunofluorescence microscopy images were acquired using a 63x objective (Plan-Apochromat, 1.4 Oil DIC, Zeiss) on an Axio Observer widefield fluorescence microscope, equipped with ApoTome.2, an Axiocam 506 mono camera and a Colibri 7 LED light source (all Zeiss). Images were processed with the zen blue software (Zeiss).

#### Cilia quantifications

Percentage of cells with primary cilia: determined manually from >100 cells in 15 fields of view for 3 biological replicates.

Cilia length: measured using Zen blue software (Zeiss) with graphics tools from >50 uninfected or infected cells in >10 fields of view and 3 biological replicates.

### Cilia assays

#### Generation of ciliated cells

hTERT-RPE-1, NIH3T3 and IMCD3 cells were grown in 6-well plates or on coverslips to 70% confluency and then serum starved for 48 hours to induce cilia formation. The percentage of cells with primary cilia was quantified by manual counting, and cilia length was determined using the Zen blue software.

#### Cilia disassembly assay

Ciliated cells, generated as described above, were either mock infected or infected with *Chlamydia*, followed by incubation in the absence of serum until the indicated time points. For serum re-addition, which served as a positive control, ciliated cells were incubated in medium supplemented with 10% FBS for 30 hours followed by fixation.

#### Cilia assembly/Formation assay

hTERT-RPE-1cells were infected with *C*.*trachomatis* as described above, followed by incubation in serum-free DMEM for 30 hours to induce cilia formation.

### Genome copy assay

The number of chlamydiae per host cell was measured by qPCR. Crude total DNA extracts were prepared by collecting RPE-1 cells (serum starved, transfected with scrambled, AurA or HDAC6-specific siRNA) and infected) in 500 μL of sonication buffer [1 mM EDTA, 10 mM Tris-HCl (pH7.5), 0.1% SDS] followed by freeze-thaw at −80°C. The crude extract was sonicated briefly for 30s at an amplitude of 20 before diluting to 1:2,000. The copy number of two chlamydial genomic loci (*euo* and *fliF* promoter regions) and two host genomic loci (*gapdh* and *ywhaz*) was quantified by qPCR (see S1 Table for primer sequences). Chlamydiae per host cell were measured by calculating *E*_h_^Ct(host cell^)/*E*_c_^Ct(chlamydia^) (*E*_h_: PCR efficiency of host genomic locus, *Ct(host cell)*: Ct value for host genomic locus, *E*_c_: PCR efficiency for *C*. *trachomatis* genomic locus, Ct(chlamydia): Ct value for *C*. *trachomatis* genomic locus).

### Progeny assay

Progeny assays were performed as previously described [46]. hTERT-RPE-1 WT or GFP-SMO Cep290 WT/KO cells treated with inhibitors or siRNAs were infected with *Chlamydia* for 30 hours at an MOI of 3. They were then washed with PBS and lysed in SPG buffer using glass beads or a one cycle freeze-thaw. The lysates were collected in microcentrifuge tubes and subjected to rigorous vortexing. Serial dilutions of the lysates in SPG were used to re-infect a fresh HeLa cell monolayer in a 96-well plate by centrifugation for 1 h at 700 x g. At 27 hpi, cells were fixed in ice-cold methanol followed by visualization of chlamydial inclusions by immunofluorescence microscopy with mouse anti-MOMP antibodies. The number of inclusions, determined over 10 fields of view using a 20X objective, was used to calculate the total number of infectious progeny (IFU/mL). The number of infectious progeny was normalized to samples treated with DMSO or scrambled siRNA as negative controls.

### Electron microscopy

RPE-1 cells transfected with scrambled, AurA or HDAC6-specific siRNA were infected with *C*.*trachomatis*. At 30 hpi, cells were washed with 1× PBS and detached from the plate using trypsin, centrifuged at 300 × *g* for 4 min, and fixed with 2% paraformaldehyde/2.5% glutaraldehyde (Polysciences Inc., Warrington, PA, USA) in 100 mM sodium cacodylate buffer, pH 7.2 for 2 h at room temperature. Processing and imaging of the samples were performed by the Washington University Center for Cellular Imaging facility.

### EdU labeling experiment

The Thermo Click-iT EdU Cell Proliferation Kit was used. In brief, serum starved RPE-1 cells that were either incubated with serum-free medium, infected with *Chlamydia* or incubated with serum-containing medium were incubated with 10 μM EdU (final concentration) for two hours prior to fixation and staining according to the manufacturer’s protocol. Following completion of the Click-iT protocol, additional antibody labeling was performed as previously stated. The percentage of EdU-positive cells was determined by microscopy analysis.

### Lysate preparation and western blot analysis

Cells were harvested by direct lysis in 2% SDS Lysis Buffer (50mM Tris pH 7.5, 150mM NaCl, 2% SDS) supplemented with 325 U mL^-1^ of Benzonase (Sigma-Aldrich) in 100 μl/well of a 6-well dish and heated to 95°C. Equal volumes of sample were loaded and resolved by SDS-PAGE. Proteins were transferred onto nitrocellulose membranes, blocked in 5% milk in PBST (5% dry powdered milk, 0.1% Tween-20, 1X PBS) and incubated with primary antibodies for 1 h at room temperature followed by 3 PBST washes. The membrane was then incubated with LI-COR IRDye conjugated secondary antibodies in blocking buffer for 1 hour. Blots were washed 4 times with PBST and imaged on the LI-COR Odyssey SA infrared imaging system (LI-COR).

### Statistical analysis

Statistical analysis of data was performed with software present in GraphPad Prism 9.0, including the unpaired two-tailed t test, the 1-way ANOVA and the 2-way ANOVA.

## Supporting information

S1 Fig *Chlamydia*-induced cilia disassembly is not specific for the cell line, method of cilia induction and *C*.*trachomatis* serovar. (A) *Chlamydia* causes primary cilia disassembly in other cell lines. Same as in Fig 1C, except for the use of Hela (left), IMCD3 (middle) and NIH3T3 (right) cells instead of RPE-1 cells. NIH3T3 and IMCD3 cells were serum starved for 48 hours, but HeLa cells were serum starved for 96 hours. Cells were mock infected or subjected to infection with *C*.*trachomatis* L2 or serum re-addition. At 30 hpi, cells were fixed and processed for immunofluorescence analysis. The percentage of cells with primary cilia is shown. (B) *Chlamydia* infects cycling as well as growth-arrested cells. RPE-1 cells, grown in DMEM in the presence (serum) or absence of serum (serum starvation), were infected with *C*.*trachomatis* L2 and processed for immunofluorescence analysis with antibodies to MOMP to detect the chlamydiae at 30 hpi. The percentage of cells with a detectable inclusion is shown. (C) Uninfected and infected cells with representative primary cilia at each time point of this time course analysis are shown. Scale bar: 5μm. (D) *Chlamydia* causes primary cilia disassembly in contact-inhibited cells. Mouse embryonic fibroblasts were grown to high confluency in the presence of serum to produce a condition of contact inhibition. These cells were then left untreated, mock-infected or infected with *Chlamydia* for 30 hours. Cells were fixed and processed for immunofluorescence analysis. The percentage of cells with primary cilia is shown. (E) RPE-1 cells, grown in DMEM containing 1% serum, were subjected to infection with *C*. *trachomatis* serovar D infection. At 72 hpi, cells were fixed and processed for immunofluorescence analysis. The percentage of cells with primary cilia is shown. For each graph in S1 Fig, three independent biological replicates were performed. Data is presented as mean ± SEM. Data were analyzed with the one-way ANOVA with multiple comparisons for (B) and (C), and the unpaired t test for (D). ns: non-significant, ****P<0.0001, *** *P*<0.001, ** *P*<0.01.
(TIF)

S2 Fig Aurora A and HDAC6 are efficiently depleted by siRNA. (A) Total cell lysates from RPE-1 cells transfected with scrambled or AurA siRNA were subjected to Western blot analysis with antibodies to AurA and GAPDH as a loading control. Quantification of Aurora A protein level relative to scrambled siRNA-treated cells are shown. (B) Total cell lysates from RPE-1 cells transfected with scrambled or HDAC6 siRNA were subjected to Western blot analysis with antibodies to HDAC6 and GAPDH as a loading control. Quantification of HDAC6 protein level relative to scrambled siRNA-treated control cells are shown. Data are presented as mean ± SEM (n = 3). An unpaired t-test was performed to compare Aurora A and HDAC6 protein levels normalized to GAPDH. ****P<0.0001. (C) RPE-1 cells were transfected with either scrambled or HDAC6-specific siRNA for 48 hours in serum-free medium, followed by either infection with *C*.*trachomatis* L2 or serum re-addition. At 30 hpi, cells were fixed and processed for immunofluorescence microscopy with antibodies to the ciliary axoneme (Arl13b) and the basal body (Cep164). DNA was visualized with the DNA dye NucBlue. Dashed lines indicate chlamydial inclusions. Insets show magnified views of the boxed regions. Scale bars: 10 μm. A quantification of this experiment is shown in Fig 2C.
(TIF)

S3 Fig Calmodulin is necessary for *Chlamydia*-induced cilia disassembly. (A) RPE-1 cells, grown in serum-free medium for 48 hours, were either treated with DMSO or calmidazolium (CMZ, 5nM) for 2 hours prior to infection with *C*.*trachomatis* L2 or serum re-addition. After 30 hours of incubation in absence or presence of CMZ, cells were fixed and processed for immunofluorescence microscopy with antibodies Arl13b to stain the ciliary axoneme (green), γ-tubulin to detect the basal body (red), and NucBlue to visualize the nucleus (blue). Dashed lines indicate chlamydial inclusions. Insets show magnified views of the boxed regions. Scale bar: 10 μm (main image). (B) Quantification of the images in A. The percentage of cells with primary cilia is shown, and three independent biological replicates were performed. Data is presented as mean ± SEM. That data were analyzed with a one-way ANOVA. ns: non-significant, ****P<0.0001. (C) RPE-1 cells, grown in serum-free medium for 48 hours, were treated with DMSO or calmidazolium (CMZ, 5nM) for 2 hours prior to infection with *C*. *trachomatis* L2. After 30 hours of incubation in absence or presence of CMZ, progeny assays were conducted to determine the number of infectious progeny. Fold changes in progeny between DMSO and inhibitor-treated cells are shown. Progeny was compared using an unpaired two-tailed t test. ****P<0.0001.
(TIF)

S4 Fig *Chlamydia* infection does not affect primary cilia formation. (A) Schematic representation of the experimental design. (B) RPE-1 cells, grown in DMEM containing 10% FBS, were simultaneously infected with *C*.*trachomatis* L2, shifted to serum-free medium and treated with DMSO or tubastatin A. At 30 hpi, cells were fixed and processed for immunofluorescence microscopy. Dashed lines indicate the location of chlamydial inclusions. Insets show magnified views of the boxed regions. Scale bar: 10 μm. (C) Quantification of the images in B. The percentage of cells with primary cilia is shown. Three independent biological replicates were performed. Data is presented as mean ± SEM. Unpaired two-tailed t test was performed. *** *P*<0.001.
(TIF)

S5 Fig Cells without cilia support a productive *Chlamydia* infection. (A) left: RPE-1 WT and Cep290 KO cells, grown in serum-free medium for 48 hours, were subjected to *C*.*trachomatis* L2 infection. At 30 hpi, cells were fixed and processed for immunofluorescence microscopy with antibodies Arl13b to stain the ciliary axoneme (green), Cep164 to detect the basal body (red) and NucBlue to visualize the nucleus (blue). Dashed lines indicate the chlamydial inclusions. Insets show magnified views of the boxed regions. Scale bar: 10 μm. right: The percentage of WT and Cep290 KO RPE-1 cells with chlamydial inclusions is shown. (B) RPE-1 WT and Cep290 KO cells, grown in serum free medium for 48 hours, were infected with *C*.*trachomatis* L2. At 30 hpi, cells were lysed, and lysates were used for secondary infection to determine the number of infectious progeny through progeny assays. The percentage of infectious progeny relative to control is shown. Data are presented as mean ± SEM (n = 3). An unpaired t-test was performed to compare the percentage of infectious progeny in RPE-1 WT vs RPE-1 Cep290 KO cells.
(TIF)

S6 Fig C.*trachomatis* infection promotes cell cycle re-entry that is prevented by blocking the AurA-HDAC6 pathway. (A) A schematic representation of the experimental design is shown. (B) Serum-starved RPE-1 cells with cilia were incubated in either serum-free medium, infected with *Chlamydia* at MOI 3 or incubated with medium with 10% serum. After 34 hours, cells were labeled with EdU for 2 hours using click chemistry (shown in white) and then processed for immunofluorescence microscopy with antibodies to Arl13b (ciliary axoneme) and Cep164 (basal body). DNA was labeled with the DNA dye NucBlue. Representative images are shown for each condition. (C) A quantification of the percentage of EdU-positive cells from the immunofluorescence images in (B) is shown. (D) Serum-starved RPE-1 cells with cilia were incubated in either serum-free medium, infected with *Chlamydia* at MOI 3 or incubated with medium containing 10% serum. At 36 hpi, cells were fixed and processed for immunofluorescence microscopy with antibodies to the Ki-67 proliferation marker. The percentage of Ki-67-positive cells are shown. (E) Same as in (B), but 2 hours prior to the infection, cells were treated with tubastatin A or PHA-680632. The quantification of the percentage of EdU-positive cells is shown. For each graph in S6 Fig, three independent biological replicates were performed. Data is presented as mean ± SEM. A 1-way ANNOVA with multiple comparisons was performed for (C) and (E) and a 2-way ANOVA with multiple comparisons was performed for (D). ns: non-significant, ****P<0.0001, *** *P*<0.001, ** *P*<0.01.
(TIF)

S1 Table Primers used for genome copy quantification. The sequences of the primers that were used for genome copy quantification by qPCR are shown.
(XLSX)

S1 Data Raw data of all figures. These numbers were used to generate all graphs of this manuscript.
(XLSX)
