# Supplementary figures and images for: Chlamydia trachomatis induces disassembly of the primary cilium to promote the intracellular infection
Source: PLoS Pathog. 2024 Jun 17;20(6):e1012303. doi: 10.1371/journal.ppat.1012303 (PMC11213297; doi:10.1371/journal.ppat.1012303)

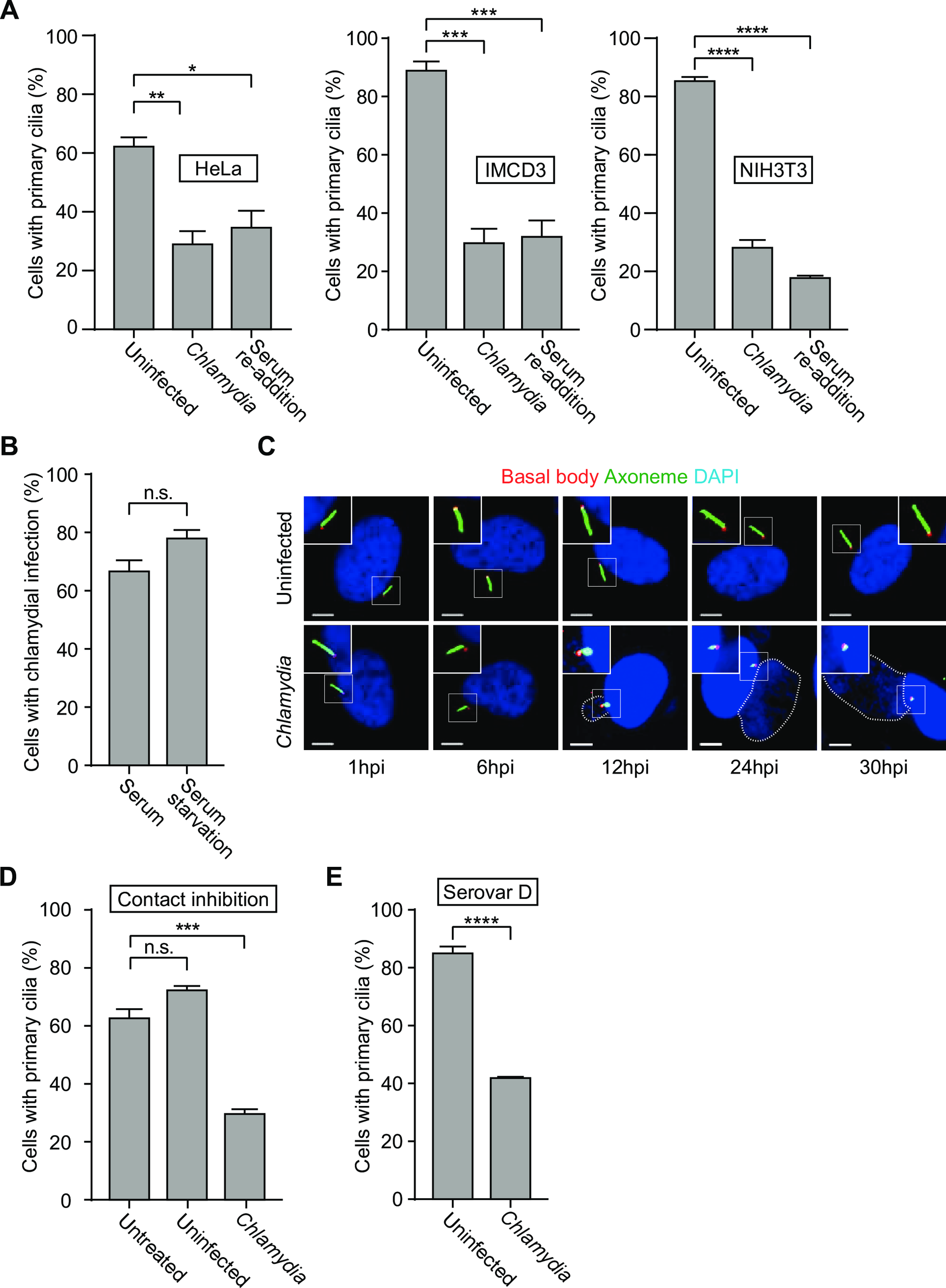

Supplement: S1 Fig — (A) Chlamydia causes primary cilia disassembly in other cell lines. Same as in Fig 1C, except for the use of Hela (left), IMCD3 (middle) and NIH3T3 (right) cells instead of RPE-1 cells. NIH3T3 and IMCD3 cells were serum starved for 48 hours, but HeLa cells were serum starved for 96 hours. Cells were mock infected or subjected to infection with C.trachomatis L2 or serum re-addition. At 30 hpi, cells were fixed and processed for immunofluorescence analysis. The percentage of cells with primary cilia is shown. (B) Chlamydia infects cycling as well as growth-arrested cells. RPE-1 cells, grown in DMEM in the presence (serum) or absence of serum (serum starvation), were infected with C.trachomatis L2 and processed for immunofluorescence analysis with antibodies to MOMP to detect the chlamydiae at 30 hpi. The percentage of cells with a detectable inclusion is shown. (C) Uninfected and infected cells with representative primary cilia at each time point of this time course analysis are shown. Scale bar: 5μm. (D) Chlamydia causes primary cilia disassembly in contact-inhibited cells. Mouse embryonic fibroblasts were grown to high confluency in the presence of serum to produce a condition of contact inhibition. These cells were then left untreated, mock-infected or infected with Chlamydia for 30 hours. Cells were fixed and processed for immunofluorescence analysis. The percentage of cells with primary cilia is shown. (E) RPE-1 cells, grown in DMEM containing 1% serum, were subjected to infection with C. trachomatis serovar D infection. At 72 hpi, cells were fixed and processed for immunofluorescence analysis. The percentage of cells with primary cilia is shown. For each graph in S1 Fig, three independent biological replicates were performed. Data is presented as mean ± SEM. Data were analyzed with the one-way ANOVA with multiple comparisons for (B) and (C), and the unpaired t test for (D). ns: non-significant, ****P<0.0001, *** P<0.001, ** P<0.01. (TIF) [file ppat.1012303.s001.tif]

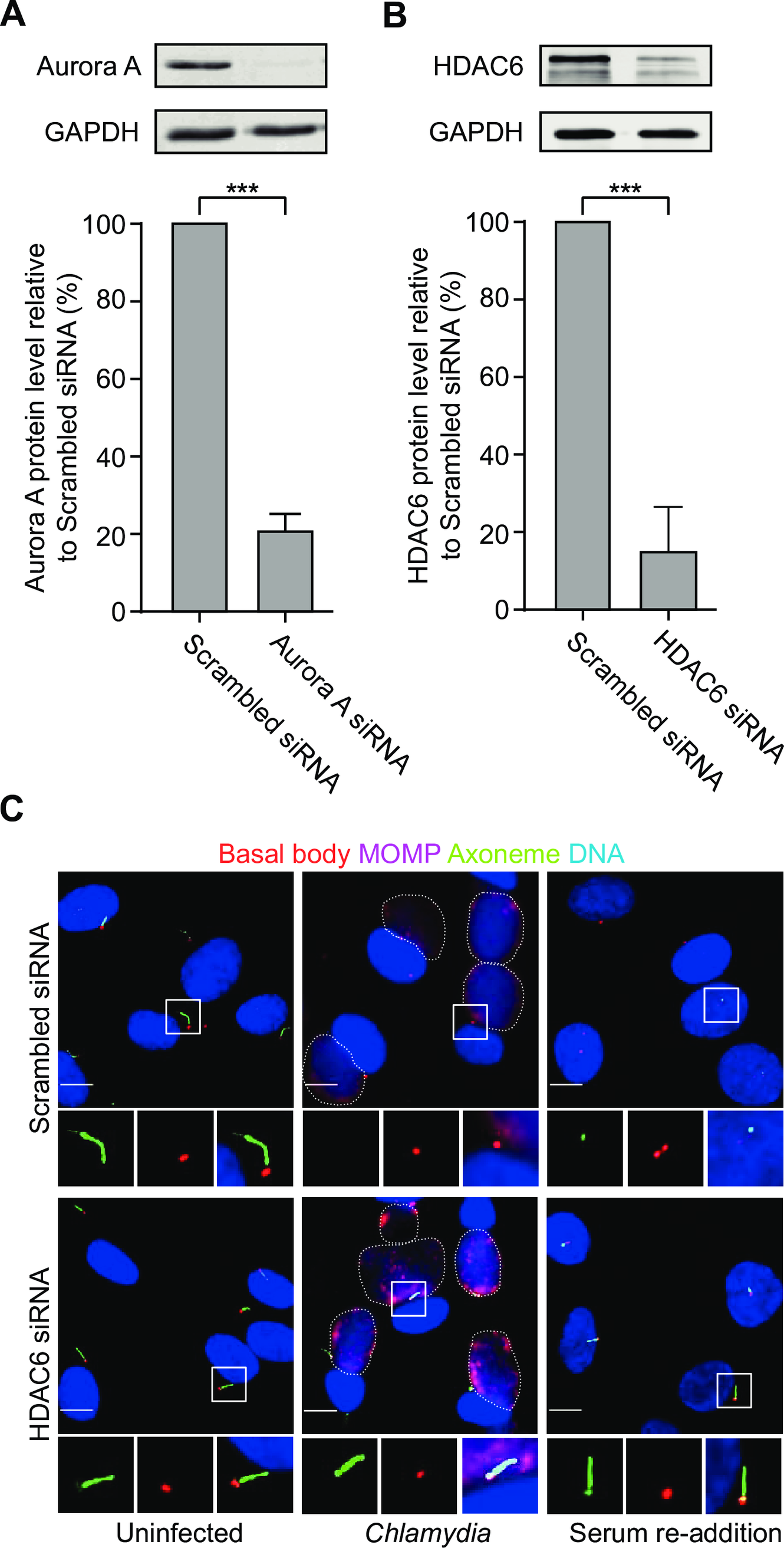

Supplement: S2 Fig — (A) Total cell lysates from RPE-1 cells transfected with scrambled or AurA siRNA were subjected to Western blot analysis with antibodies to AurA and GAPDH as a loading control. Quantification of Aurora A protein level relative to scrambled siRNA-treated cells are shown. (B) Total cell lysates from RPE-1 cells transfected with scrambled or HDAC6 siRNA were subjected to Western blot analysis with antibodies to HDAC6 and GAPDH as a loading control. Quantification of HDAC6 protein level relative to scrambled siRNA-treated control cells are shown. Data are presented as mean ± SEM (n = 3). An unpaired t-test was performed to compare Aurora A and HDAC6 protein levels normalized to GAPDH. ****P<0.0001. (C) RPE-1 cells were transfected with either scrambled or HDAC6-specific siRNA for 48 hours in serum-free medium, followed by either infection with C.trachomatis L2 or serum re-addition. At 30 hpi, cells were fixed and processed for immunofluorescence microscopy with antibodies to the ciliary axoneme (Arl13b) and the basal body (Cep164). DNA was visualized with the DNA dye NucBlue. Dashed lines indicate chlamydial inclusions. Insets show magnified views of the boxed regions. Scale bars: 10 μm. A quantification of this experiment is shown in Fig 2C. (TIF) [file ppat.1012303.s002.tif]

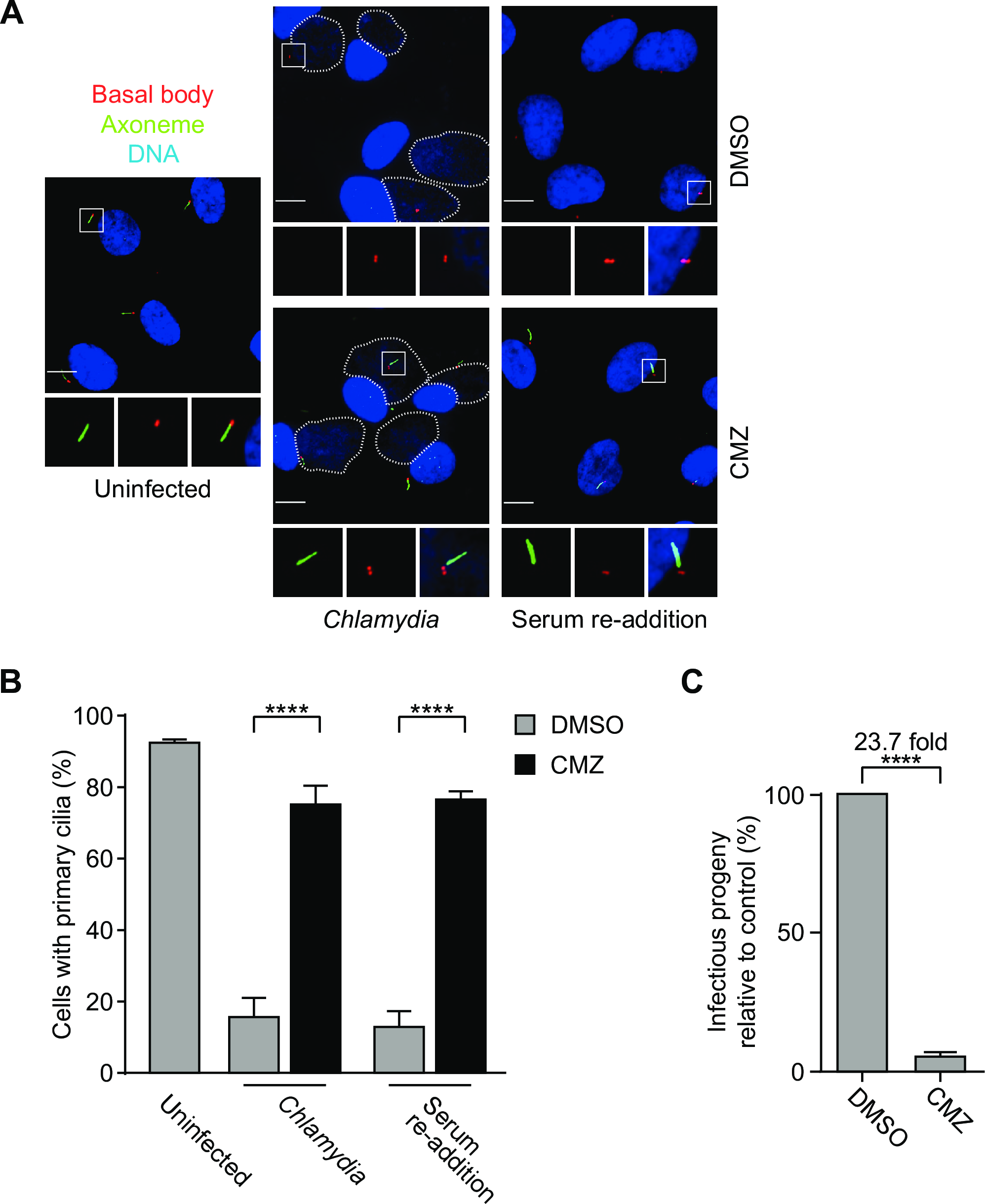

Supplement: S3 Fig — (A) RPE-1 cells, grown in serum-free medium for 48 hours, were either treated with DMSO or calmidazolium (CMZ, 5nM) for 2 hours prior to infection with C.trachomatis L2 or serum re-addition. After 30 hours of incubation in absence or presence of CMZ, cells were fixed and processed for immunofluorescence microscopy with antibodies Arl13b to stain the ciliary axoneme (green), γ-tubulin to detect the basal body (red), and NucBlue to visualize the nucleus (blue). Dashed lines indicate chlamydial inclusions. Insets show magnified views of the boxed regions. Scale bar: 10 μm (main image). (B) Quantification of the images in A. The percentage of cells with primary cilia is shown, and three independent biological replicates were performed. Data is presented as mean ± SEM. That data were analyzed with a one-way ANOVA. ns: non-significant, ****P<0.0001. (C) RPE-1 cells, grown in serum-free medium for 48 hours, were treated with DMSO or calmidazolium (CMZ, 5nM) for 2 hours prior to infection with C. trachomatis L2. After 30 hours of incubation in absence or presence of CMZ, progeny assays were conducted to determine the number of infectious progeny. Fold changes in progeny between DMSO and inhibitor-treated cells are shown. Progeny was compared using an unpaired two-tailed t test. ****P<0.0001. (TIF) [file ppat.1012303.s003.tif]

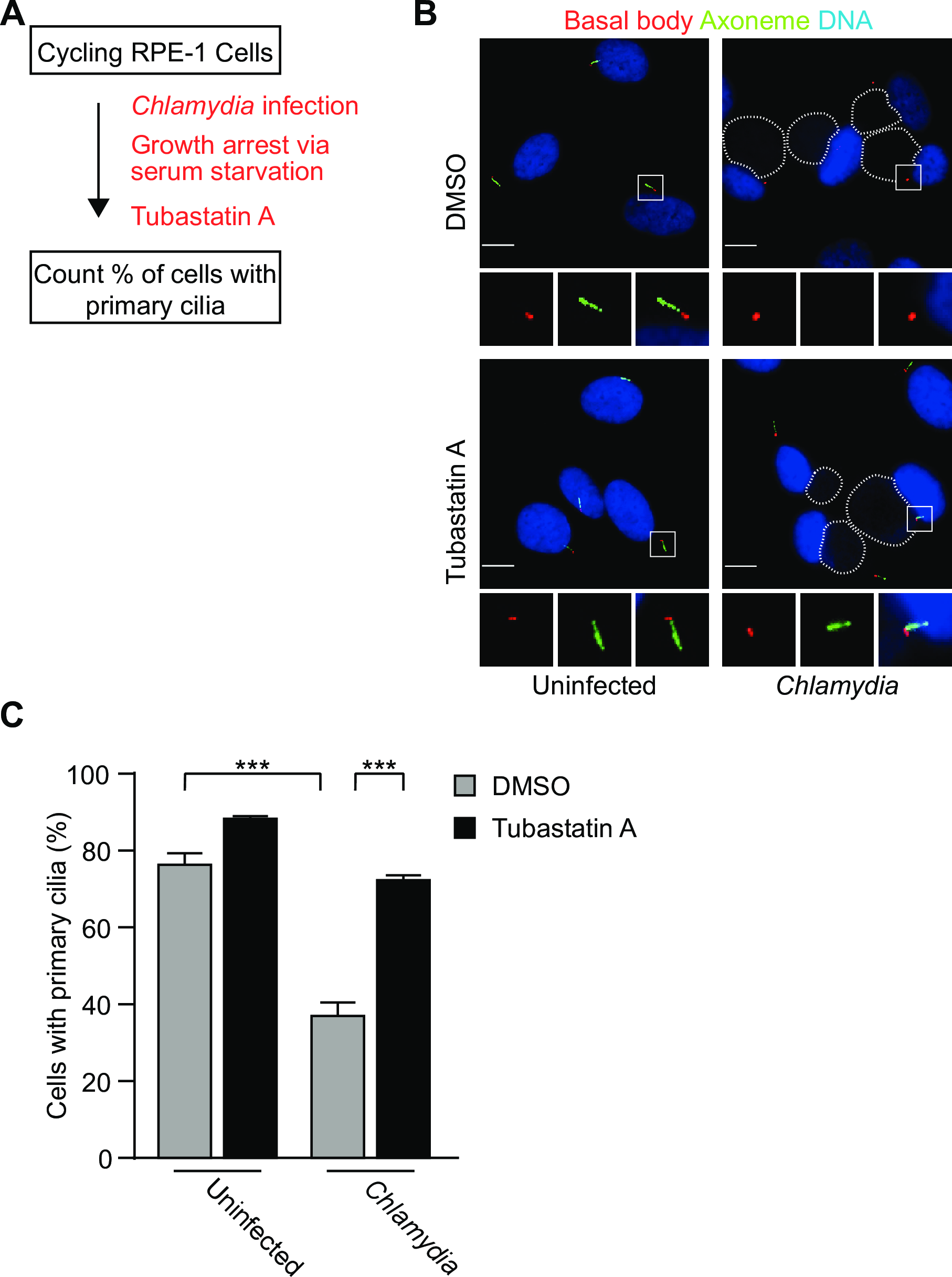

Supplement: S4 Fig — (A) Schematic representation of the experimental design. (B) RPE-1 cells, grown in DMEM containing 10% FBS, were simultaneously infected with C.trachomatis L2, shifted to serum-free medium and treated with DMSO or tubastatin A. At 30 hpi, cells were fixed and processed for immunofluorescence microscopy. Dashed lines indicate the location of chlamydial inclusions. Insets show magnified views of the boxed regions. Scale bar: 10 μm. (C) Quantification of the images in B. The percentage of cells with primary cilia is shown. Three independent biological replicates were performed. Data is presented as mean ± SEM. Unpaired two-tailed t test was performed. *** P<0.001. (TIF) [file ppat.1012303.s004.tif]

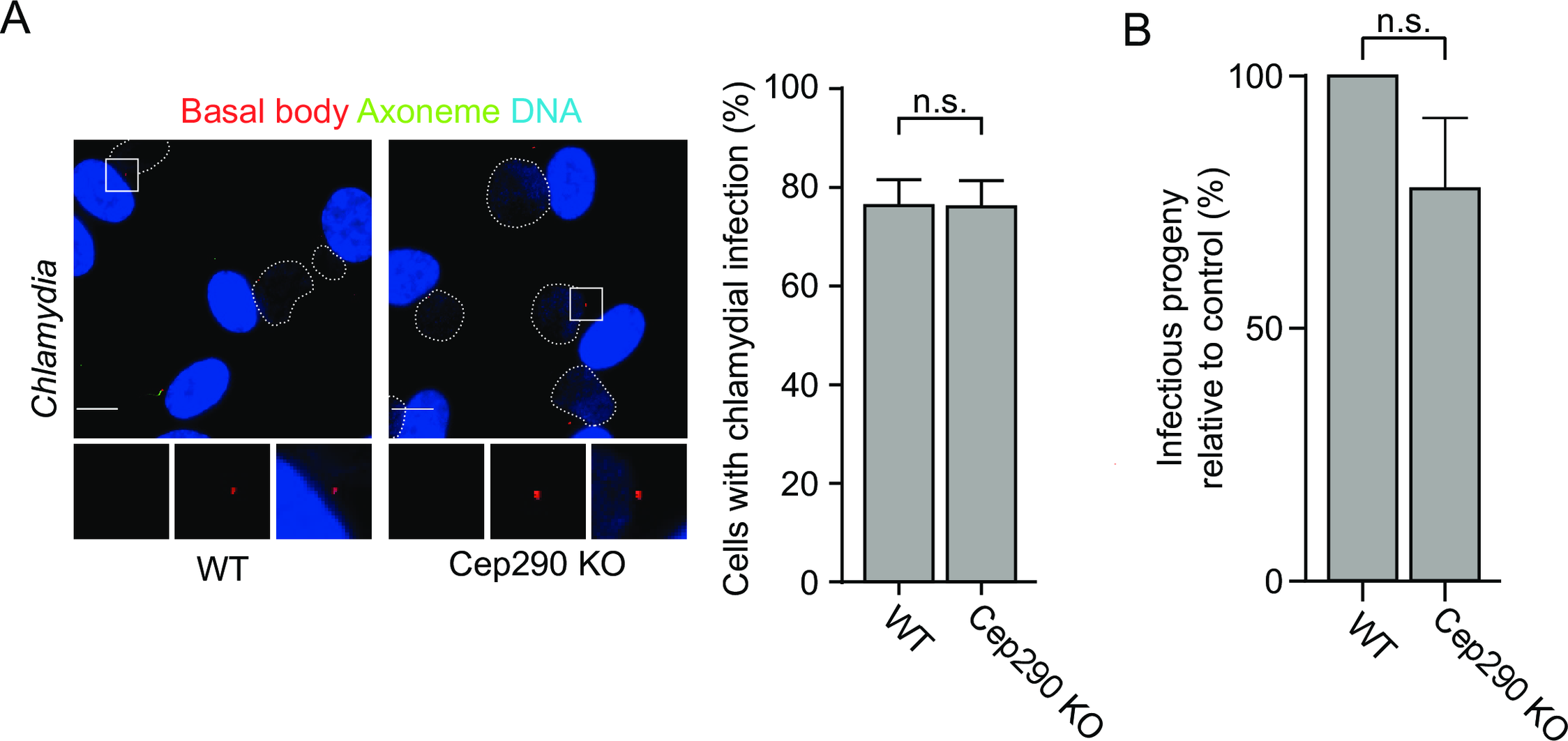

Supplement: S5 Fig — (A) left: RPE-1 WT and Cep290 KO cells, grown in serum-free medium for 48 hours, were subjected to C.trachomatis L2 infection. At 30 hpi, cells were fixed and processed for immunofluorescence microscopy with antibodies Arl13b to stain the ciliary axoneme (green), Cep164 to detect the basal body (red) and NucBlue to visualize the nucleus (blue). Dashed lines indicate the chlamydial inclusions. Insets show magnified views of the boxed regions. Scale bar: 10 μm. right: The percentage of WT and Cep290 KO RPE-1 cells with chlamydial inclusions is shown. (B) RPE-1 WT and Cep290 KO cells, grown in serum free medium for 48 hours, were infected with C.trachomatis L2. At 30 hpi, cells were lysed, and lysates were used for secondary infection to determine the number of infectious progeny through progeny assays. The percentage of infectious progeny relative to control is shown. Data are presented as mean ± SEM (n = 3). An unpaired t-test was performed to compare the percentage of infectious progeny in RPE-1 WT vs RPE-1 Cep290 KO cells. (TIF) [file ppat.1012303.s005.tif]

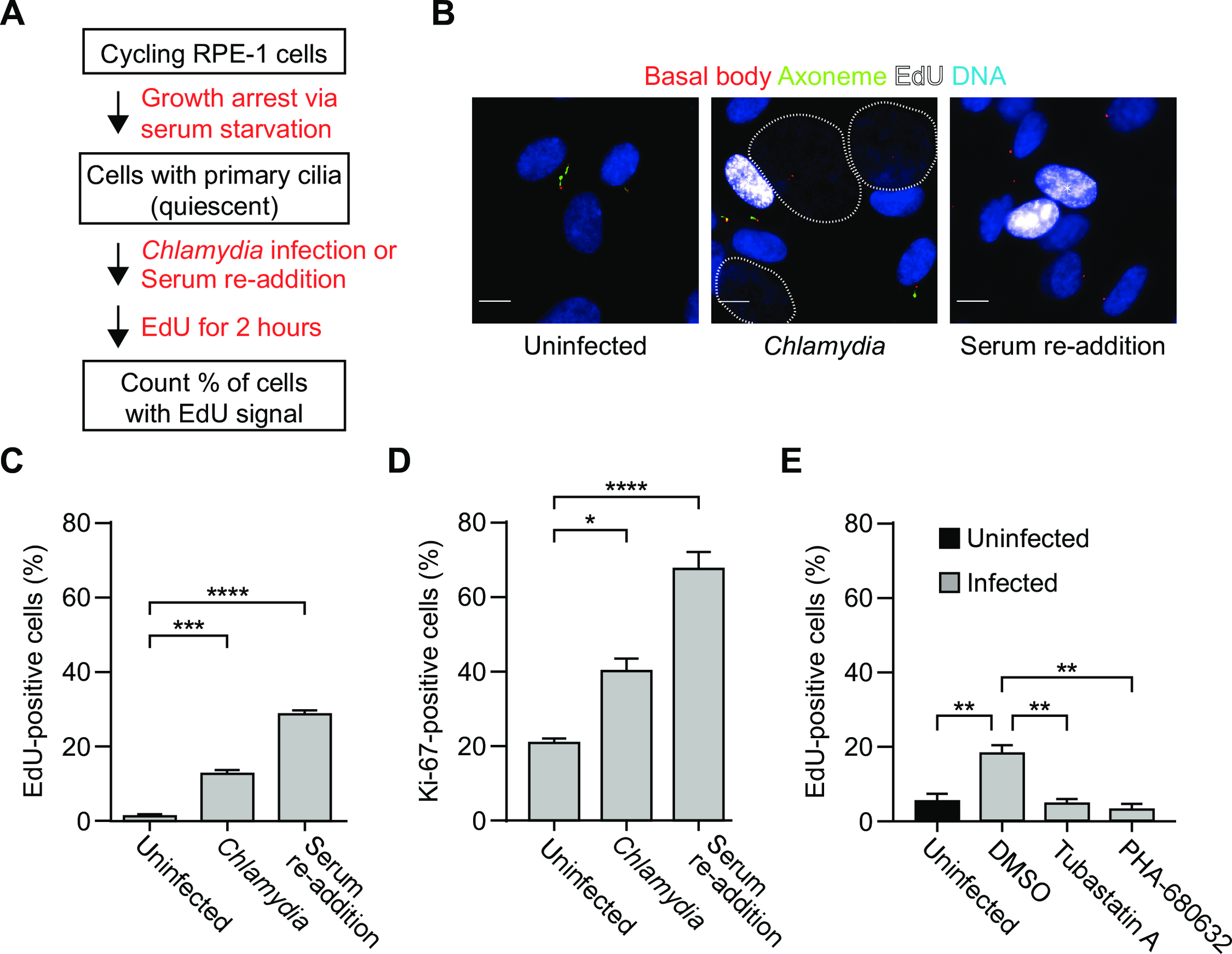

Supplement: S6 Fig — (A) A schematic representation of the experimental design is shown. (B) Serum-starved RPE-1 cells with cilia were incubated in either serum-free medium, infected with Chlamydia at MOI 3 or incubated with medium with 10% serum. After 34 hours, cells were labeled with EdU for 2 hours using click chemistry (shown in white) and then processed for immunofluorescence microscopy with antibodies to Arl13b (ciliary axoneme) and Cep164 (basal body). DNA was labeled with the DNA dye NucBlue. Representative images are shown for each condition. (C) A quantification of the percentage of EdU-positive cells from the immunofluorescence images in (B) is shown. (D) Serum-starved RPE-1 cells with cilia were incubated in either serum-free medium, infected with Chlamydia at MOI 3 or incubated with medium containing 10% serum. At 36 hpi, cells were fixed and processed for immunofluorescence microscopy with antibodies to the Ki-67 proliferation marker. The percentage of Ki-67-positive cells are shown. (E) Same as in (B), but 2 hours prior to the infection, cells were treated with tubastatin A or PHA-680632. The quantification of the percentage of EdU-positive cells is shown. For each graph in S6 Fig, three independent biological replicates were performed. Data is presented as mean ± SEM. A 1-way ANNOVA with multiple comparisons was performed for (C) and (E) and a 2-way ANOVA with multiple comparisons was performed for (D). ns: non-significant, ****P<0.0001, *** P<0.001, ** P<0.01. (TIF) [file ppat.1012303.s006.tif]
